# Using *Drosophila melanogaster* as a suitable platform for drug discovery from natural products in inflammatory bowel disease

**DOI:** 10.3389/fphar.2022.1072715

**Published:** 2022-12-05

**Authors:** Minghui Xiu, Yixuan Wang, Dan Yang, Xueyan Zhang, Yuting Dai, Yongqi Liu, Xingyao Lin, Botong Li, Jianzheng He

**Affiliations:** ^1^ College of Public Health, Gansu University of Chinese Medicine, Lanzhou, China; ^2^ Provincial-level Key Laboratory for Molecular Medicine of Major Diseases and the Prevention and Treatment with Traditional Chinese Medicine Research in Gansu Colleges and University, Gansu University of Chinese Medicine, Lanzhou, China; ^3^ Key Laboratory of Dunhuang Medicine, Ministry of Education, Lanzhou, China; ^4^ College of Basic Medicine, Gansu University of Chinese Medicine, Lanzhou, China

**Keywords:** inflammatory bowel disease, *Drosophila melanogaster*, natural products, drug discovery, molecular pathways

## Abstract

Inflammatory bowel disease (IBD) is a chronic and life-treating inflammatory disease that can occur in multiple parts of the human intestine and has become a worldwide problem with a continually increasing incidence. Because of its mild early symptoms, most of them will not attract people’s attention and may cause more serious consequences. There is an urgent need for new therapeutics to prevent disease progression. Natural products have a variety of active ingredients, diverse biological activities, and low toxicity or side effects, which are the new options for preventing and treating the intestinal inflammatory diseases. Because of multiple genetic models, less ethical concerns, conserved signaling pathways with mammals, and low maintenance costs, the fruit fly *Drosophila melanogaster* has become a suitable model for studying mechanism and treatment strategy of IBD. Here, we review the advantages of fly model as screening platform in drug discovery, describe the conserved molecular pathways as therapetic targets for IBD between mammals and flies, dissect the feasibility of *Drosophila* model in IBD research, and summarize the natural products for IBD treatment using flies. This review comprehensively elaborates that the benefit of flies as a perfact model to evaluate the therapeutic potential of phytochemicals against IBD.

## Introduction

Inflammatory bowel disease (IBD) is a chronic, progressive, life-long disease that leads to bowel damage and disability, including Crohn’s disease (CD) and ulcerative colitis (UC) (Salas et al., 2020). In recent years, the incidence of IBD has generally increased in many countries around the world, and is closely related to genetic susceptibility, environmental factors and dysbiosis, but it also brings great economic and social pressure (Guzzo et al., 2022). To date, IBD is not easy to completely cured, which encourages researchers to investigate more effective therapeutics for this disease (Che et al., 2022; Zilbauer, 2022). At present, some immunosuppressants, 5-aminosalicylic acid, and steroids have been clinically used to alleviate patients’ syndromes. However, they have serious adverse reactions in patients, such as anemia, diarrhea, and glaucoma (Zhang et al., 2021). Therefore, the development of effective and safer drugs for IBD treatment are urgently needed.

Most of research objects on drug screening and evaluation are model organisms, such as cells, *C. elegans*, *Drosophila*, zebrafish, mammals (Mccammon and Sive, 2015; Maitra and Ciesla, 2019). Model organisms are essential for investigating the pathogenesis and drug screening for human diseases. Cell culture is often used model for drug screening, but the drug toxicity reactions in the screening process cannot fully reflect the body tissue-specific responses. Although mammal models have provided crucial materials for the study of pathogenesis, pathological process and the mechanisms underlying drug-related behaviors, they are not ideal. This is mainly due to the expensive and long-term experimentations, breeding and ethical implications. Recently, *Drosophila* has been proved as an excellent model organism for dissecting the mechanism and drug library screening, such as cancer, aging, nociception, neurodegenerative diseases. Until now, *Drosophila* as a model helps researchers get the Nobel Prize in Physiology or Medicine for six times (Kitani-Morii et al., 2021). The advantages of fly are small size, genetic amenability, low-cost maintenance, and excellent genetic and molecular tools. Meanwhile, fly has a high homology with human at the organ and gene level (Maitra and Ciesla, 2019). These classcial advantages provide great opportunities for researchers to investigate the mechanism of IBD and drug discovery research (Apidianakis and Rahme, 2011; Su, 2019; Madi et al., 2021).

The pathogeny of IBD is very complicated and has not been completely understood. Disruption of intestinal homeostasis is closely related to the occurrence and development of IBD. Many signaling pathways related with IBD such as JAK/STAT, Wnt/Wg, Nrf2/Keap1, TLR4/NF-κB, Notch pathways were identified in flies, and are conserved in humans (Hu et al., 2021; Yang et al., 2022). Various natural products have shown that various natural molecules or herbal extractions are widely applied in the prevention and treatment of IBD in various animal models (Yang et al., 2022). Consistently, the similar function of natural products treating intestinal inflammatory diseases are found in flies and mammals (Pereira et al., 2017). In this article, we discussed the advantages of fly model as screening platforms in drug discovery, and described the conserved modelcular pathways as therapetic targets for IBD in fly and mammal. Nextly, we dissected the feasibility of *Drosophila* model in IBD research and summarized the natural products for IBD treatment in fly model.

## Use of *Drosophila* model as screening platforms in drug discovery

**FIGURE 1:**
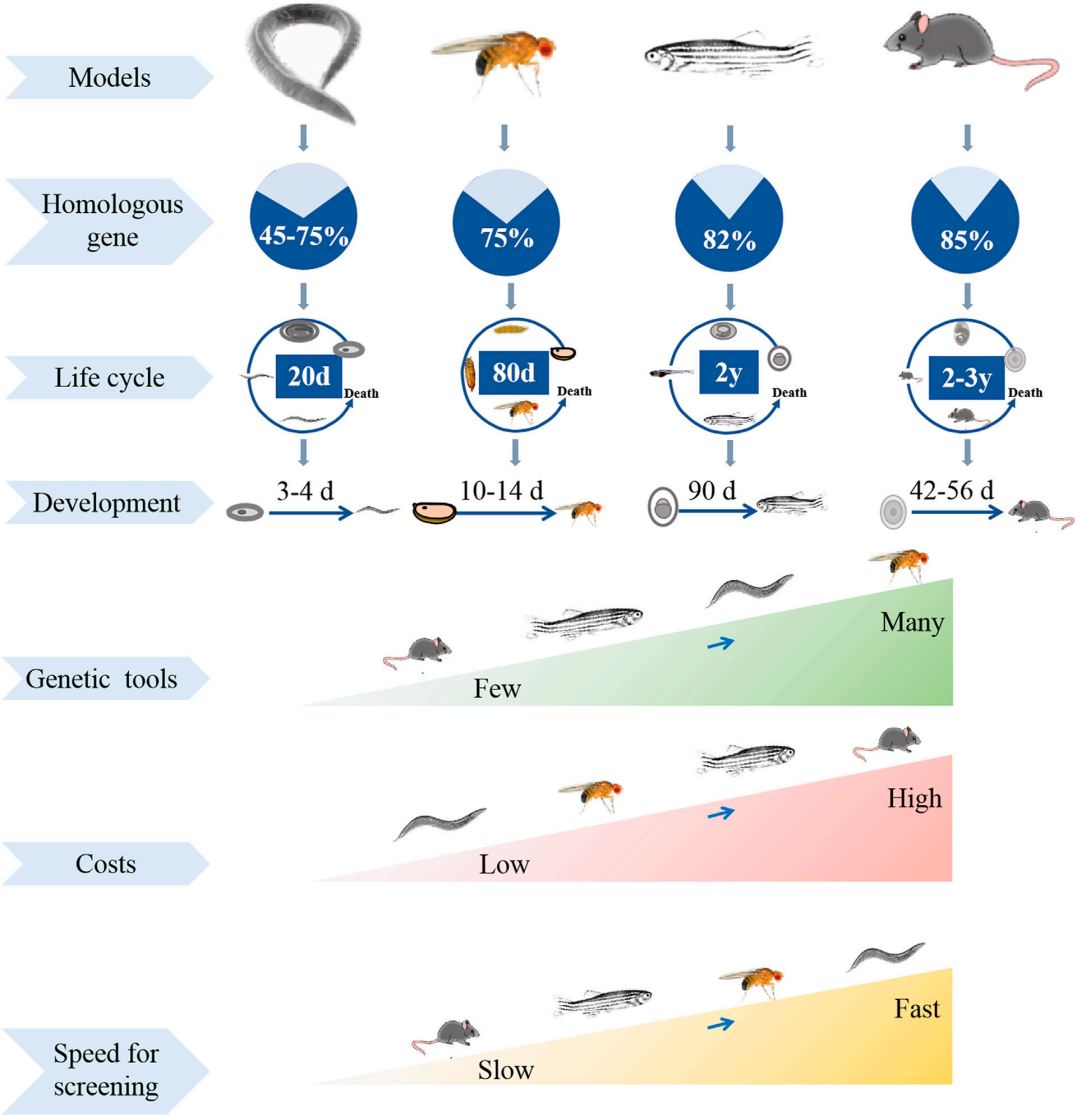
Comparison of experimental models in different species in multiple aspects.

Screening thousands of drug candidates need to speed various time and money, and often leads to uncertain success. At present, many models are used for screening potential drugs, such as cells, yeast, *C. elegans*, *D. melanogaster* and mammals, in which some can accelerate the process of drug discovery, when some are easy to collect valuable data (Figure 1). High-throughput screening of cell cultures is one of the most widely used methods for potential drug screening (Maitra and Ciesla, 2019). However, cell culture belongs to drug administration experiments *in vitro*, and the drug toxicity reactions in the screening process cannot fully reflect the body tissue-specific responses. Unbiased drug experiments using appropriate model organisms *in vivo* enable rapid and specific screening of drug candidates with therapeutic potential (Willoughby et al., 2013). Rodents such as rats and mice are the most common models for drug screening, but they often result in economic and ethical pressures, also have low reproductive rates and long lifespan (Bilen and Bonini, 2005). An ideal drug-screening model should be highly manipulable while reflecting human biology (Hergovich et al., 2006). Fruit fly has been universally used by researchers to investigate genetics and human diseases, such as neurodeneration, cancer, and nociception (Hwang and Lu, 2013; Kitani-Morii et al., 2021; Chiang et al., 2022; He et al., 2022). Compared to cell culture model, fruit fly is a complex “whole animal” model with organs and tissue systems functioning synergistically. Fly can be administered in a variety of ways, and its behavioral activity can be easily monitored to analyze the therapeutic and toxic effects of drugs (Shahzad et al., 2021). Compared to rodents, fly is relatively economical and easy to manipulate, and it has short generation time, large collections of transgenetic strains, and less ethical concerns (Hwang and Lu, 2013). Therefore, *Drosophila* is an ideal model for economical and rapid large-scale screening of therapeutically useful natural products.

## The advantages of genetic manipulation

The key reason why fly can serve as a classic biological model is its highly conserved molecular pathways and powerful molecular tools that easily manipulate the expression of specific genes (Senturk and Bellen, 2018). Fly gene sequencing in 2000 shows that many basic physiological and functional characteristics are highly conserved between flies and humans, meanwhile about 75% genes of human-related diseases are homologous in flies (Bier, 2005). Homologues or orthologues of human genes in flies are easily knocked in or knocked out using genetic tools to mimic specific disease-associated phenotype. One of the widely used genetic tools is the GAL4/UAS system. GAL4 as a yeast-derived transcription factor bind to Upstream Activating Sequence (UAS), driving the downstream gene expression (Takano-Shimizu-Kouno and Ohsako, 2018). Many strains that express GAL4 can target diverse tissues, specific cells and given genes. The progeny of crosses between the targeted GAL4 and UAS strains are used to analyze the function. UAS targeted RNA interference (RNAi) or green fluorescent protein (GFP) combines with GAL4 drive to suppress specific gene expression or label fluorescent marker in any tissue or cell, which is beneficial for studying various organ and tissue diseases (Pagliarini and Xu, 2003; Weasner et al., 2017; Xie et al., 2018). This system is widely used to label specific intestinal cells and regulate signaling in intestinal cells in flies. For example, Escargot (Esg) as a specific marker for enteroblasts and intestinal stem cells (ISCs) can generate esg-Gal4; UAS-GFP reporter flies, in which the enteroblasts and ISCs are marked as GFP. External stimulation or infection significantly enhance stem cells proliferation followed by intensity of GFP increasing (Micchelli and Perrimon, 2006; Buchon et al., 2009). Drice is a negative regulator of Imd signaling and is required for intestinal homeostasis. When esg-Gal4 driver flies cross with UAS targeted Drice-RNAi flies, the offspring has decreased Drice expression in ISCs. Developed from this technique is the temporal and regional gene expression targeting (TARGET) system, in which temperature sensitive GAL4-inactivating protein GAL80 could repress GAL4 activity at permissible temperatures, which is beneficial for precise temporal control of transgene expression (Mcguire et al., 2004). The FLP recombinase/FLP recognition target (FLP/FRT) system is also commonly used to regulate gene expression or induce somatic recombination in homologous chromosomes of flies (Theodosiou and Xu, 1998). In addition, other tools such as CRISPR-Cas9 and Cre/LoxP that developed in mammalian system have also been used in flies (Nakazawa et al., 2012; Bier et al., 2018). The availability of these genetic tools makes flies as a favorable model for potential drug screening.

## The advantages of phenotype-based research

Most of the drug discovery efforts carried out in flies begin with phenotype-based research, and the related phenotypes are simple and easy to detect, and reliable conclusions can be drawn in a short time (Giacomotto and Segalat, 2010; Maitra et al., 2022). For example, various neurodegenerative diseases exhibit slowness of locomotor ability and loss of a specific subset of neurons. The locomotor ability in flies is monitored by the negative geotaxis climbing test. The specific subset of neurons can be easily marked as fluorescence by using genetic methods, and are monitored by microscopy techniques (Maitra and Ciesla, 2019). Eye degeneration in Alzheimer (AD) and Parkinson (PD) transgenic fly models is used as a tool for pharmacological screening (He et al., 2021). Survival assays in flies are used to determine the role of potential drugs on lifespan, stress resistance and developmental defects (Dai et al., 2020). Simple feeding assays are used to investigate the therapeutic effect of drug candidates (Yang et al., 2022). In addition, survival assays, development and reproduction assays in flies are used to evaluate the toxicity of drug candidates and determine the optimal drug concentrations. Importantly, with easily observable phenotypes associated with gut diseases, flies have significant advantages for discovering drugs that treat IBD disease (Maitra and Ciesla, 2019). For example, the intestinal length is easily measured; integrity of the intestinal epithelial barrier is evaluated by using the “smurfs” experiments; the midgut digestive function is characterized by the gastrointestinal acid-base homeostasis (Sheng Q. et al., 2021).

Thus, using *Drosophila* model to screen natural drugs will help to overcome the limitiations of cell culture assays regarding toxicity and pharmacological assessment, and will also quickly reduce the scope from huge potential drug candidates. In addition, fly can be widely used to dissect the mechanism of functional compounds on disease pathogenesis.

### Conserved molecular pathways as therapetic targets for intestinal inflammatory disease

The intestinal epithelium is the first line of defense in the digestive tract against pathogens entering the body, and maintains the intestinal homeostasis. Intestinal homeostasis in flies is regulated by evolutionarily conserved molecular pathways, such as JAK/STAT, Nrf2/Keap1, TLR4/NF-κB, Wnt/Wg and Notch signling pathways. An imbalance among these types of pathways in epithelium could result in IBD.

### JAK/STAT pathway

The Janus kinase/signal transducer and activator of transcription (JAK/STAT) signaling pathway is a transport hub that transduces cues from extracellular cytokines into transcriptional changes in the nucleus, which participates in many cellular processes, such as cell growth, differentiation and migration of immune cells (O'Shea et al., 2013). The inappropriate activation or delection of JAK/STAT pathway is associated with inflammatory and autoimmune diseases, including IBD, Parkinson’s disease (PD) and psoriasis (Xin et al., 2020). Inhibition of this pathway can suppress multiple cytokine pathways in the treatment of IBD. JAK is a key intracellular signaling mediator in IBD, which transduces signals from cytokine receptors on the cell surface to the nucleues, and its dysregulation leads to the pathological process of IBD (Dudek et al., 2021). Presently, several JAK inhibitors are used to treat IBD patients (Wang L. et al., 2021). Tofacitinib as a JAK inhibitor is clinically used for UC patients, and various other inhibitors such as filgotinib, TD-1473 and upadacitinib are currently being investigated in preclinical and clinical trials (Salas et al., 2020; Harris and Cummings, 2021). In addition, STAT is the final effector of JAK-STAT signaling pathway (Moon et al., 2021). Some STAT inhibitors have also been studied in treating IBD, although no clinical trials have been conducted in patients with IBD (Kasembeli et al., 2018) Various plant-derived natural compounds such as curcumin, ellagic acid and paeonol have been proved to alleviate IBD by affecting the JAK-STAT pathway in IBD animal models (Marin et al., 2013; Yang et al., 2013; Moon et al., 2021). Thus, therapeutic intervention of the JAK-STAT pathway can efficiently regulate the complex inflammation driven by diverse inflammatory cytokines in IBD.

The JAK/STAT pathway in flies has the same essential signaling components as in mammals (Herrera and Bach, 2019). When enterocytes (ECs) in fly midgut are subjected to stress signaling mediated by apoptosis, chemical injury, or pathogen infection, pro-inflammatory ligands (Upd, Upd2, Upd3) are rapidly produced and released. These ligands activate one receptor Domeless (Dome), leading to the activation of one JAK and one STAT transcription factor, termed Hopscotch (Hop) and Stat 92E, respectively. The pathway activity is downregulated by Socs36E in a negative-feedback loop. Socs36E is a suppressor of cytokine signaling protein. Core components of the JAK-STAT pathway in flies are homologous to interleukin 6 (IL-6), the JAK2 and STAT5 in mammals (Myllymaki and Ramet, 2014). The JAK/STAT pathway plays an important role in fly midgut homeostasis and tissue regeneration following various challenges, such as bacterial infection, directed cell ablation or stress signaling (Buchon et al., 2009). Under normal conditions, JAK/STAT pathway facilitates the rapid proliferation and differentiation of ISCs to drive epithelial regeneration (Jiang et al., 2009). The over-activation of JAK/STAT pathway causes excessive proliferation of ISCs and abnormal differentiation of EC cells, which disrupts the balance of intestinal homeostasis, and promotes the deterioration of intestinal epithelium (Herrera and Bach, 2019).

### Nrf2/Keap1 pathway

Nuclear factor-erythroid-derived 2-related factor 2 (Nrf2), a member of the basic-region leucine zipper (bZIP) transcription factor, is one of the most important regulators of the cell defense system against oxidative stress and inflammatory damage (Mou et al., 2020). Nrf2 regulates the transcription of more than 200 genes, including antioxidant proteases and inflammatory regulators, by binding antioxidant response elements (AREs) in the promoter region (Raghunath et al., 2018). The activity of Nrf2 is negatively mediated by Kelch-like ECH-associated protein 1 (Keap1) that is a protein rich in cysteine (Sekhar et al., 2010). Various studies have shown that activation of the Keap1-Nrf2-ARE signaling pathway can provide protection against various stress and inflammation-related diseases, including IBD (Cuadrado et al., 2018; Piotrowska et al., 2021). Previous studies found that DSS-induced Nrf2 knockout mice had higher expression of colonic inflammatory markers and cytokines, and more severe colonic injury compared to control colitis mice (Cheung et al., 2014). Administration of Nrf2 activator dimethyl fumarate (DMF) alleviated DSS-induced experimental colitis in mice (Li et al., 2020). The activator of Nrf2, 5-aminosalicylic acid, has been used clinically in the treatment of IBD (Kang et al., 2017; El-Baz et al., 2020). Meanwhile, various plant-derived natural compounds have been demonstrated to alleviate IBD by affecting the Keap1-Nrf2-ARE pathway in animal model systems of IBD, such as luteoline (Li et al., 2016), curcumin (Lin et al., 2019) and Flos puerariae extract (Yang et al., 2022) Therefore, the Nrf2 activator is considered as a potential drug for the treatment of IBD.

Nrf2 is highly homologous to CncC in *Drosophila* (Loboda et al., 2016). There are three isoforms of Cnc: CncA, CncB and CncC, of which CncC plays an important role in the oxidative stress process in flies (Pomatto et al., 2017). The mechanism of the oxidative stress response in flies is similar to that in mammals. Under non-stress conditions, CncC activity is restricted by dKeap1 (Sykiotis and Bohmann, 2008). When flies are under oxidative stress and intestinal damage, electrophile and ROS interrupt the interaction between CncC and Keap1. CncC forms a heterodimer with Maf-S in the nucleus, binds to the ARE and activates transcription of the target gene (Misra et al., 2013). Nrf2 can promote intestinal homeostasis by specifically controlling the proliferation activity of ISCs. Loss of Nrf2 in ISCs led to accumulation of ROS and accelerated degeneration of the intestinal epithelium (Hochmuth et al., 2011).

### TLR4/NF-κB pathway

TLR4/NF-κB is an important inflammatory signaling transduction pathway, which closely participates in cell differentiation and proliferation, apoptosis, and pro-inflammatory response (Yu et al., 2022). Toll-like receptors (TLRs) play an important role in recognizing invading microbial pathogens and leading to innate immune response for the host defense, and also involved in the pathogenesis of IBD (Frantz et al., 2018; Lu et al., 2018). As one class of TLRs, TLR4 is the first characterized TLR in the mammalian, and mainly regulates the intestinal inflammation. The expression of TLR4 significantly increases in the intestinal epithelium of patients with active UC (Toiyama et al., 2006). Nuclear factor kappa B (NF-κB) is the final transcription factor of the TLR4 pathway. Upon activation, NF-κB dimers translocate to the nucleus, and promotes the transcription and translation of inflammatory mediators, which results in the development of intestinal diseases in mammals (Chen et al., 2018). Many components of natural products such as apigenin, luteolin and hesperidin have been proven to ameliorate intestinal inflammation by inhibiting the TLR4 receptor activation and blocking the nuclear translocation of NF-κB in mammals (Guazelli et al., 2021; Zuo et al., 2021; Begum et al., 2022; Zhang et al., 2022). Thus, downregulation of the TLR4/NF-κB pathway is a potential therapeutic strategy against IBD.

Toll signaling pathway is first identified in *Drosophila* (Lye and Chtarbanova, 2018). The first identification of TLRs in 1988 and then subsequent recognition of its one homolog called TLR4 in humans in 1997 (Hashimoto et al., 1988; Medzhitov et al., 1997). Activation of Toll in flies results in the formation of a signaling complex containing the adaptor proteins MyD88, Tube and the kinase Pelle *via* a homotypic TIR interaction (Tauszig-Delamasure et al., 2002). This complex indirectly promotes the NF-κB-like transcription factors Dif and Dorsal to the nucleus, leading to the expression of cytokines and antimicrobial peptides (AMPs) (Lamiable et al., 2016; He et al., 2022). Intestinal epithelial cells have the evolutionarily conserved TLR pathway in flies and mammals (Ferguson and Foley, 2022). Toll signaling in flies plays a role in the maintance of gut homeostasis *via* regulating the balance between microbe-induced epithelial cell damage and stem cell repair (Buchon et al., 2009).

### Wnt/Wg pathway

Wnt signaling pathway is an important pathway for the maintenance of stem cells, which controls cell proliferation, impacts the cell cycle and regulates the self-renewal of some tissues in mammals (Nusse and Clevers, 2017; Liu et al., 2022). Wnt signaling pathway regulates the stem cell proliferation, differentiation and migration in the intestinal epithelium, and participates in the pathogenesis of IBD (Flanagan et al., 2018). Decreased the Paneth cell alpha-defensin is one of the factors in IBD pathogenesis (Khoramjoo et al., 2022). Diminished the Wnt pathway transcription factor (Tcf-4) expression could weaken enteric antimicrobial defense by reducing the Paneth cell alpha-defensin (Pu et al., 2021; Khoramjoo et al., 2022). In addition, studies have shown that in Tcf-4 knockout mice, reduced level of Paneth cell alpha-defensin in intestine permitted bacteria to invade the epithelium and result in colitis (Wehkamp et al., 2007). Inhibition of Wnt signaling pathway could disrupt the intestinal-stem-cell homeostasis, consequently leading to intestinal diseases in mammals (Kuhnert et al., 2004; Perochon et al., 2018). Various natural molecules, such as Astragaloside IV and procyanidin, have been reported to promote mucosal healing and alleviate colitis symptoms by activating the Wnt pathway in mice (Pu et al., 2021). Thus, it is worthwhile to increase the window of opportunities for IBD treatment by activating Wnt pathway.

Fly and mammalian guts not only have similar morphology, but also share the same Wnt signaling pathway. The *Drosophila* genome encodes seven Wnt genes including Wingless (Wg), Wnt2, Wnt4, Wnt5, Wnt6, Wnt10, and WntD (Tian et al., 2018). Only Wg and Wnt4 are expressed in the fly midgut (Perochon et al., 2018). Wnt pathway plays an important role in the self-renewal of the fly gut. When flies are exposed to damage from chemical toxins, bacterial infection and mechanical stress, the expression level of Wg protein increases in EBs of the midgut epithelium, leading to compensatory ISC proliferation and differentiation to re-establish homeostasis (Jiang et al., 2016; Liu et al., 2017). Meanwhile, the inhibition of Wnt signaling in the intestinal epithelium abolishes gut regeneration (Jiang et al., 2016; Liu et al., 2017).

### Notch pathway

Notch signaling is a highly conserved cell-cell communication pathway. It regulates the development and differentiation of cells, tissue function, organs formation, and maintains the homeostasis of the body through interactions between adjacent cells (Artavanis-Tsakonas et al., 1999; Herbert and Stainier, 2011). Notch signaling pathway is critically linked to the pathogenesis of several diseases such as IBD, cancer, and autoimmune diseases (Okamoto et al., 2009). In the intestine, Notch pathway regulates the secretory of intestinal cells such as Paneth cells and goblet cells. Increased Notch pathway leads to a deficiency of Paneth cells, and ultimately induces a collapse of the intestinal barrier in patients with IBD (Gersemann et al., 2011). Activated γ-secretase promotes the generation of Notch intracellular domain (NICD) (Kumar et al., 2016). Aberrant expression of NICD leads to decrease in the quantity of goblet cells in patients with UC (Zheng et al., 2011). Consequently, studies have shown that the γ-secretase inhibitor dibenzoazepine alleviates IBD by suppressing the Notch pathway (Shinoda et al., 2010). *L. acidophilus* could regenerate goblet cells by inhibiting Notch transcriptional program factors to alleviate Salmonella-induced-colitis in mice (Wu et al., 2018). Thus, inhibiting Notch pathway is considered to be an effective strategy in the treatment of IBD.

The Notch gene is first named in flies in the 1910s (Stubbs et al., 1990). Most essential components of the Notch signaling pathway are conserved between flies and humans (Yang et al., 2022)*.* Notch pathway participates in regulating the self-renewal and differentiation of ISCs. In adult *Drosophila* intestines*,* the Notch ligand Delta is specifically expressed in ISCs (Ohlstein and Spradling, 2007). Upon division of the ISC, Delta promotes the expression of Notch target genes by activating the Notch receptor in its sister cell (Fre et al., 2011). Notch pathway is closely associated with gut homeostasis. Under normal conditions, Notch pathway promotes ISCs to replenish the loss of EE and EC to maintain intestinal homeostasis (Fre et al., 2011). Ingestion of chemicals or pathogenic bacteria could disrupt stem cell differentiation and midgut homeostasis by activating Notch pathway in the *Drosophila* intestine (Kux and Pitsouli, 2014).

## The feasibility of *Drosophila* model in IBD research

The *Drosophila* fly has been demonstrated to be an excellent model for dissecting the mechanisms of intestinal disease, due to its similar anatomical features with mammal intestine, and its genetic and functional conservation with mammals (Lin and Hackam, 2011; Medina et al., 2022). A suitable method for investigating the pathogenesis of human IBD and screening candidate drugs from natural products is to produce animal model of IBD, including fly, zebrafish, and rodents.

### Conserved structure of midgut between fly and mammalian

**FIGURE 2:**
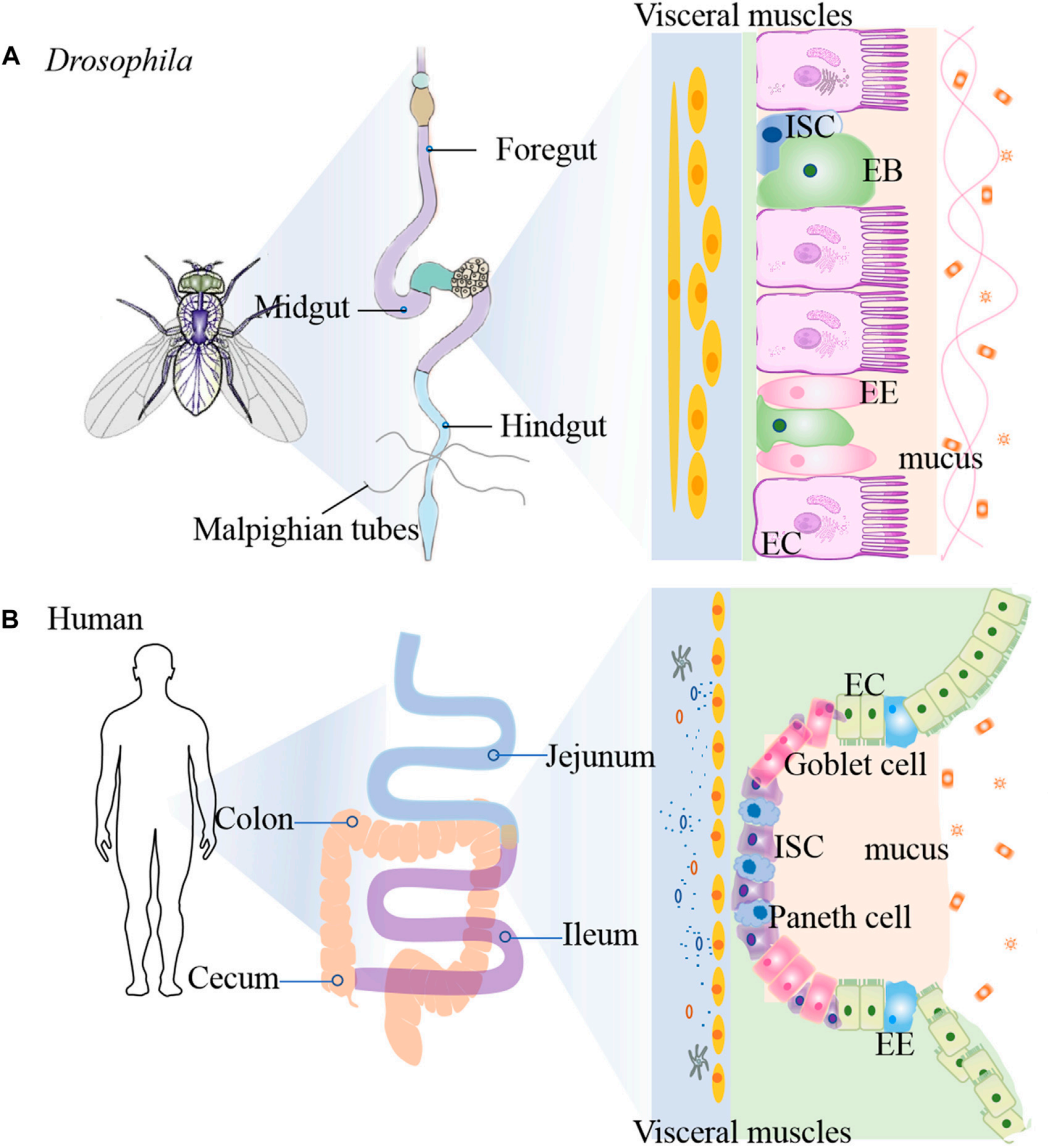
Comparison of intestinal tract anatomy between adult *Drosophila melanogaster* and human. The adult *Drosophila* **(A)** and human **(B)** intestinal tracts share structural and functional homology.

The gastrointestinal (GI) tract is a first layer of defense against the various microbes. The fly GI tract is the tissue of digestion and absorption, and shares many properties with the mammalian counterparts, including the stomach, small intestine, and colon (Figure 2) (Liu et al., 2017). Fly midgut has emerged as an attractive system to investigate the intestinal inflammatory disease due to not only the cell lineage of this tissue is simple and well-defined, but also it shows similarites to the mammalian intestine (Jiang and Edgar, 2012). The flies intestine is composed of three main regions: foregut, midgut and hindgut (Lemaitre and Miguel-Aliaga, 2013). The foregut encompasses pharynx, oesophagus and crop, which is an organ involved in food storage. The midgut extends from the cardia to the junction with the hindgut, while the Malpighian tubules connect to the gut. The hindgut fulfills the excretory functions of the fly gastrointestinal system, which is similar with the mammal large intestine (Miguel-Aliaga et al., 2018). The copper cell region (CCR) is located approximately in the middle of the midgut and is acidic similar to the mammalian stomach (Strand and Micchelli, 2013). The posterior midgut is the most metabolically active and immune responsive region of the fly gut and is similar with the mammal small intestine, where the hindgut corresponds to the mammal colon (Micchelli and Perrimon, 2006; Capo et al., 2019).

The epithelium of the fly midgut and mammal gut contains uniform ISCs that undergo division and asymmetric cell fate decision (Liu et al., 2017). The adult *Drosophila* gut is composed of an epithelial monolayer consisting of 4 cell types: intestinal stem cells (ISCs), absorptive enterocytes (ECs), enteroblasts (EBs) and secretory enteroendocrine (EE) cells (Medina et al., 2022). Each ISC divides symmetrically into two ISCs or asymmetrically into an renewed ISC and EB. EBs differentiate into diploid EEs or polyploid ECs (Chen et al., 2018). Similarly, ISCs self-renew and differentiate into the transit amplifying cells in mammals, and then proliferate and differentiate into secretory cells and ECs, and dedicate Paneth cell progenitors (Liu et al., 2017). ISCs are characterized by expression of high levels of cytoplasmic Delta-rich vesicles, triggering Notch signaling in neighboring EBs (Ohlstein and Spradling, 2007). Su(H)Gbe-lacZ as a transcriptional reporter of Notch signaling is used as EB cell marker (Micchelli and Perrimon, 2006). The enhancer trap fly snail family gene escargot (esg) targets both ISC and EB (Micchelli and Perrimon, 2006). Brush border Myosin (MyolA) marks the ECs and Prospero (Pros) marks the EEs (Jiang et al., 2009). Chemicals such as dextran sulfate sodium (DSS) and sodium dodecyl sulfate (SDS), or bacterial infection can damage the midgut, and also stimulate ISC proliferation (Amcheslavsky et al., 2009). Compared to mammalian stem cells, the flies possess a much simpler lineage in intestinal epithelium. However, the cellular functions and molecular principles that dictate ISC proliferation and differentiation are well conserved from flies to mammals (Medina et al., 2022). For example, JAK-STAT pathway (Xu et al., 2011), Wg/Wnt pathway (Liu et al., 2017), Hippo pathway (Ren et al., 2010) and EGFR pathway (Jiang et al., 2011). All these pathways have been implicated in human IBD. Thus, investating the role of ISC proliferation in flies will help us to find the way for human IBD mechanism.

### Intestinal inflammatory model in flies

**FIGURE 3:**
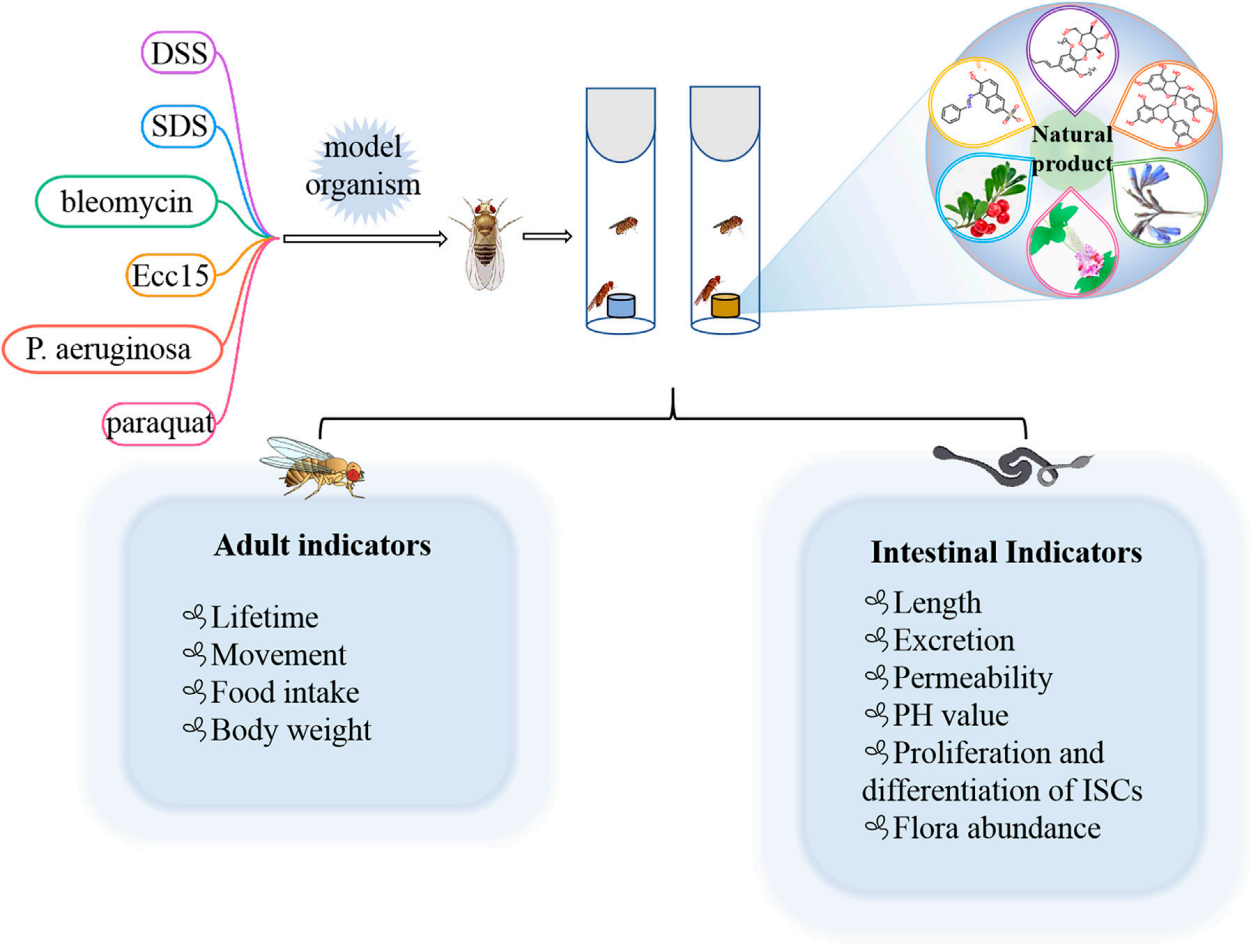
Different chemical inducers were used to construct IBD models in *Drosophila*, and effective natural products were screened by detecting corresponding indicators in adults and intestines.

Many preclinical models of IBD are currently estabolished to investigate the pathogenesis and therapy. In rodents, DSS, SDS and 2,4,6-trinitrobenzene sulfonic acid (TNBS) have been frequently employed (Yu et al., 2022; Zhang et al., 2022). Because of the high conservation with mammals, flies are also used to induce intestinal inflammation model *via* feeding DSS or SDS (Figure 3) (Lee et al., 2021; Wei et al., 2022; Yang et al., 2022). Briefly, newly ecolosed (3–5 day old) female or male flies were maintained on control diet or natural products diet for 7 days. Then flies was transferred into the empty vial containg 1% agar to starve for 2 h, flies were moved into vials containing filter papers soaked with 5% sucrose solution with or without DSS (3% or 4%) or SDS (0.5% or 0.6%), respectively. Filter papers were replaced every 2 days. For survival studies, adult flies were fed with DSS or SDS until all flies died, and number of dead flies was recorded twice per day. For intestinal morphology analysis, flies treated with DSS or SDS for 72 h were dissected in the cold PBS and immediately observed under a microscope. After flies were fed with DSS or SDS for 60 h, the intestine integrity and gastrointestinal acid-base homeostasis were investigated. Smurf assay was widely used to detect the intestine integrity, in which flies were fed with food containing a blue dye (2.5% w/v) for 12 h, fly was remarked as a Smurf when the dye coloration could be observed outside the digestive tract. The bromophenol blue assay was used to measure gastrointestinal acid-base homeostasis, in which flies were fed with 2% Brmophenol blue sodium (Sigma, B5525) for 12 h, images were captured after dissection. For observation of midgut epithelial cells, flies were fed with DSS or SDS for 72 h, then the nucleus and microvilli of midgut epithelial cells were observed by using transmission electron microscope. For dead intestinal cells detection, the dissected guts of flies fed with DSS or SDS for 72 h were stained with 7-amino-actinomycin D (7-AAD) for 30 min. The imaged were observed under confocal microscope. For reactive oxygen species assay, flies were exposed to SDS or DSS for 48 h, the intestines were dissected in cold PBS, incubated in 5 μM H2DCFDA or 5 μM dihydroethidium (DHE) for 10 min in dark environment, then washed in PBST, and immediately observed under a confocal microscope. For detecting proliferation and differentiation of ISCs, the guts of esg-GAl4; UAS-GFP flies or Dl-GAl4; UAS-GFP flies were dissected, and observed under a confocal microscope. The number of progenitor cells or ISCs was performed by counting the number of GFP positive cells per unit area.

Some of the less common agents such as bleomycin, *P. aeruginosa*, *Erwinia carotovora* 15 (Ecc15), and paraquat could also lead to gut injury in *Drosophila* (Amcheslavsky et al., 2009; Apidianakis et al., 2009; Lei et al., 2022). For example, bleomycin leaded to enterocyte-specific damage and cell loss in gut of flies, which in turn caused ISC to divide faster and facilitated enteroblast differentiation into new enterocytes (Amcheslavsky et al., 2009). Oral administration of *P. aeruginosa* and Ecc15 strains could increase the number of intestinal progenitors and induced apoptosis of mature cells to establish an intestinal injury model (Apidianakis et al., 2009; Lei et al., 2022). Overall, the current methods used to estabolish the IBD model in flies are easy and simple to operate. It is beneficial for researchers exploring the occurrence and development mechanism of IBD in human and screening the potential drugs from nature products.

## Natural products screening for IBD treatment in flies

**FIGURE 4:**
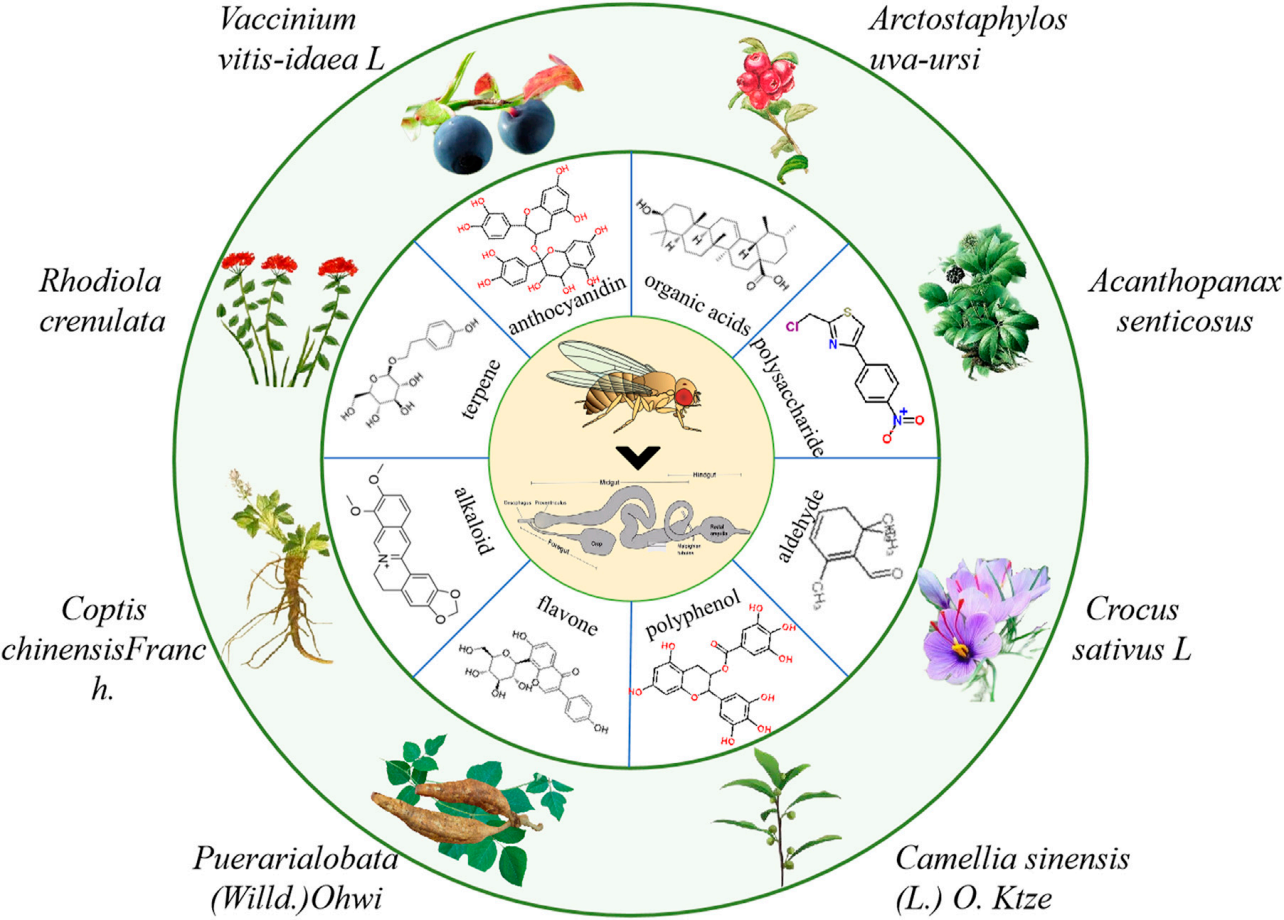
The information of partial natural products that play a crucial role in the treatment of IBD in flies.

Currently, the clinical drugs for IBD treatment are mainly synthetic compounds such as aminosalicylic acids, corticosteroids, immunosuppressants, biological agents, *etc.*, which have many side effects (Neurath, 2017). Natural products as secondary metabolite have a wide range of biological activities and a high degree of bio-availability. Their multi-component and multi-target action characteristics have unique advantages in the prevention and treatment of IBD. Until now, various natural molecules and herbal extractions have been found to treat IBD (Duan et al., 2021). Therefore, it is very important to further screen natural products that have therapeutic effects on IBD using different animial model (Ding et al., 2017; Shao et al., 2019). Here, we summarized the natural molecules and herbal extractions that have significantly protective and therapeutic effects on intestinal inflammation in flies (Figure 4).

### Natural molecules for protecting intestinal injury

Natural molecules have exhibited efficiency in protecting intestinal injury and improving symptoms in flies. For example, Acanthopanax senticosus polysaccharides (ASPS) supplementation could improve the disrupted intestinal homeostasis in flies under SDS stimulation, in which reduced the intestinal epithelial cell death, decreased ROS accumulation and antimicrobial peptide (AMP) expression (Zhang et al., 2020). Administration of ASPS also reduced the excessive ISCs proliferation and differentiation mainly by epidermal growth factor receptor (EGFR), JNK and Notch signaling pathway when flies were exposed to DSS (Lei et al., 2022). Consistently, ASPS supplementation in mice could also ameliorate LPS-induced intestinal injury, including decreased intestinal morphological deterioration, elevated the mucosal barrier and enhanced intestinal tight junction proteins expression, which mainly through inhibiting TLR4/NF-κB signaling pathway (Yasueda et al., 2020). Safranal as one of the main components of saffron significantly alleviated the DSS or Ecc15 induced intestinal epithelial cell death and excessive proliferation of ISCs to protect intestinal integrity in flies (Lei et al., 2022). This protective process was regulated through inhibition of the JAK/STAT signaling, EGFR signaling, and JNK signaling pathways in flies (Huang et al., 2022). The protective function of safranal were also reported *in vitro* and mice (Lertnimitphun et al., 2019), in which safranal supplementation decreased NO production, COX-2 and iNOS in LPS-stimulated RAW264.7 cells, and also alleviated severity of inflammation and crypt damage in the DSS-induced colitis mice. These studies elucidate that safranal may be a candidate for IBD therapy. Agar oligosaccharides (AOS) are marine prebiotics with significant anti-inflammatory effects (Ma et al., 2019). AOS supplementation alleviated the injuries of microvilli and mitochondria of gut, ameliorated the intestinal inflammation by modulating the microbiota and the gene expression of AMPs, mTOR and AMPK pathways that related with immune and cell autophagy in SDS-induced inflammatory model of flies (Ma et al., 2021). Ursolic acid (UA) is an anti-inflammatory natural triterpenoid widely distributed in various vegetables and fruits (Checker et al., 2012). UA could remarkably prevent intestine injury in SDS-stimuated flies by inhibiting ISCs hyperproliferation, decreasing excessive activation of JNK/JAK/STAT signaling pathway (Wei et al., 2022). Meanwhile, UA was found to alleviate the DSS-induced intestinal damage by reducing the upregulation of NF-κB in mice (Liu et al., 2016; Ma et al., 2021). Caffeic acid (CA) is a widespread natural phenolic small molecule, which also inhibited the dysregulation of ISCs, ameliorated the gut hyperplasia defect, and reduced aging induced mortality in flies (Sheng et al., 2021). CA could significantly attenuate the DSS-induced murine UC mainly *via* ameliorating the disease severity, loss of eptithelium and crypts, mucosal ulcerations, and secretion of inflammatory cytokines (Xiang et al., 2021). Polysaccharide from Premna microphylla turcz (PPMT) have anti-inflammatory functions *in vitro* (Li et al., 2021). In SDS-induced inflammatory flies, PPMT significantly prolonged the lifespan, reduced the rupture of microvilli and restored the nuclear structure in the midgut, and improved gene expression levels of immune-related AMP pathway, mTOR pathway and Imd pathway (Ma et al., 2021).

### Herbal extractions for protecting intestinal injury

Some herbal extractions have also been validated to have great protective function in fly model of IBD. For example, bilberry anthocyanins extracts (BANCs) have a wide range of biological activities and can be used to prevent or treat inflammation-related diseases (Farzaei et al., 2015). In DSS-induced inflammatory flies, BANCs remarkably enhanced the survival rate, restored the intestinal morphology and integrity, which mainly by modulating Nrf2 signaling pathway (Zhang et al., 2022). Consistently, BANCs could reduce intestinal inflammation in acute and chronic DSS-colitis with decreased histological scores and cytokine secretion in DSS-induced Balb/c mice (Piberger et al., 2011). Our previous studies found that Flos Puerariae extract (FPE) ameliorated the intestinal inflammation *via* modulating intestinal integrity and various signaling pathways in SDS-inflamed flies, in which FPE enhanced the survival rate, maintained intestinal morphological integrity, reduced the ISCs proliferation, and also rescued the altered expression levels of gene and protein in JAK-STAT signaling, Nrf2/Keap1 signaling and Wnt signaling pathways in the gut (Yang et al., 2022). Larvae of the *Allomyrina dichotoma* (ADL) as a high nutritional food are widely used to treat gut-related disease in China and Korea. In DSS-fed flies, oral administration of ADL extract remarkably increased the survival rate, reduced intestinal cell apoptosis and gut permeability. Meanwhile, ADL extract supplementation promoted the E-cadherin gene expression and restored the original membrane localization of DSS-disrupted E-cadherin contiguous with the armadillo (Lee et al., 2021). Rhodiola crenulata is widely used in phytotherapy in Asian countries and Eastern European (Chen et al., 2020). R crenulata extracts supplementation could prevent inflammatory diseases of the intestine in flies, in which protected against shorten intestinal length and epithelial cell death, decreased ROS levels, and increased the expression of antimicrobial peptide genes under bacterial and SDS stimulation (Zhu et al., 2014). Furthermore, the protective function of R crenulata extracts were also reported in mice. Ingestion of R crenulata extracts alleviated damage of inflammation, maintained intestinal barrier function, inhibited cell apoptosis and regulated gut microbiome in DSS-induced colitis mice (Wang et al., 2021). Extracts of *Crocus sativus L*. supplementation protected against SDS-induced intestinal damage in flies mainly *via* decreasing epithelial cell death and ROS levels in the gut (Liu et al., 2016). However, because of multiple compounds in these herbal extractions, the active ingredients and mechanisms have not been determined clearly, which need to be further explored in various animal model of IBD in future.

As mentioned above, the pharmacological function of many natural molecules and herbal extractions in treating IBD is conserved in *Drosophila* and rodents. Thus, *Drosophila* can be used as an excellent model for screening natural products for treating IBD that can be subsequently validated in a mammal system (Table 1).

**TABLE 1:** Natural products that treat IBD in fly and mice models.

Natural products	*Drosophila*		Mice		References
	Phenotype	Mechanism	Phenotype	Mechanism	
Safranal	ISCs proliferation ↓, ECs death↓, gut integrity↑ ROS↓	JNK pathway↓, EGFR pathway↓, JAK/STAT pathway↓	weight loss↓, crypt damage↓, colon length↑	MAPK pathway↓ NF-κB pathway↓	Lertnimitphun et al., (2019), Lei et al., (2022)
Caffeic Acid	survival rate↑, ISCs proliferation↓, gut integrity↑, ROS↓	JNK signaling↓	colon length↑, histopathology score↓, MDA↓,CAT↑	Nrf-2/HO-1 pathway↑	Wan et al. (2021), Sheng et al. (2021a); Zielinska et al. (2021)
Ursolic Acid (UA)	intestine integrity↑, intestine length↑, cell death↓, ROS↓,MDA↓	JNK/JAK/STAT pathway↓	colon length↑, weight loss↓, flora abundance↓	MAPK pathway↓ IL-6/STAT3 pathway↓, PI3K pathway↓	Wei et al. (2022), Sheng et al. (2021b)
Acanthopanax senticosus polysaccharide	ECs death↓, survival rate↑, gut homeostasis↑, ROS↓	EGFR pathway↓, JNK pathway↓, Notch pathway↓	villus height↑, mucosal barrier↑, occludin-1↑, ZO-1↑,TNF-α↓, PGE_2_↓	TLR4/NF-κB pathway↓	Han et al., (2016), Zhang et al., (2020)
Polysaccharide from Premna microphylla turcz (PPMT)	survival rate↑, rupture of microvilli↓, AMPs-related genes↑	Imd pathway↑, TOR pathway↑, Intestinal autophagy pathway↑	—		Song et al. (2021)
Flos Puerariae extract (FPE)	survival rate↑, intestinal integrity↑, ISCs proliferation↓	Nrf2/Keap1 pathway↑, JAK-STAT pathway↓, Wnt pathway↓	—		Yang et al. (2022)
Bilberry anthocyanins (BANCs)	survival rate↑, intestinal integrity↑, dead ISCs ↓,ROS↓	Nrf2 pathway↑	histological score↓, colon length↑, apoptotic score↓, TNF-α↓IL-6↓	—	Piberger et al., (2011), Zhang et al., (2022)
*Carthamus tinctorius L*	survival rate↑, ECs damage↓	—	intestinal integrity↑, Firmicutes/Bacteroidetes ratio↓	—	Zhou et al., (2016), Jo et al., (2017), Liu et al., (2018)
Rhodiola crenulata extract	ECs death↓, survival rate↑, ROS↓,AMP↑	—	gut permeability↓, colon length↑, ZO-1↑,occludin↑, IL-6↓,TNF-α↓	—	Zhu et al. (2014), Wang et al. (2021a)
*Larvae of the Allomyrina dichotoma (ADL)*	gut cell apoptosis↓ gut permeability↓ E-cadherin↑	—	—		Lee et al. (2021)
*Aucklandia lappa Decne*	survival rate↑, ECs damage↓	—	colon length↑, body weight↑, IL-1β↓,IL-6↓, TNF-α↓	NF-κB/MAPK pathway↓, Nrf2-Hmox-1 pathway↑	Zhou et al., 2016, Lim et al., (2020), Chen et al., 2022
*Sanguisorba officinalis L*			body weight↑, colon length↑, histopathological score↓,IL-6↓	Atg7-dependent Mφ autophagy pathway↑	Zhou et al., 2016, Yasueda et al., (2020)
*Alpinia katsumadai Hayata*			diarrhea↓, colon length↑, histological injury↓, MPO↓,TNF-α↓,IL-1β↓	TLR4 pathway↓ NLRP3 pathway↓	He et al., 2016; Zhou et al., 2016
*Salvia miltiorrhiza Bunge*			body weight↑, colon histology score↓, colon length↑	TLR4/PI3K/AKT/mTOR pathway↓	Feng et al., (2021); Peng et al., (2021)
*Raphanus sativus L*			body weight↑, colon damage scores↓, TNF-α↓,IL-1β↓	MAPK-NF-κB pathway↓	Choi et al., (2016); Zhou et al., (2016)
*Codonopsis pilosula (Franch.) Nannf (C. pilosula)*	ECs damage↓, melanotic tumor formation↓, gut length↑ AMP↑,Dpt↑, Mtk↑	Imd pathway↑	—		Zhou et al. (2016)
*Saussurea lappa (Decne.) C.B.Clarke (S. lappa)*					
*Imperata cylindrica Beauv.var.major (Nees)*					
*Melia toosendan Sied. Et Zucc. (M.toosendan)*					

## Concluding remarks and future directions

The fruit fly has been proved as an excellent model organism for investigating the mechanism and drug library screening of cancer (Gonzalez, 2013), nociception (He et al., 2022), and neurodegenerative diseases (Sneddon, 2018); Here, we have emphasized that *Drosophila* is widely used to study the molecular mechanisms of IBD and is a perfect model for high-throughput drug screening from natural products. The major advantages of flies are its sophisticated genetics, low cost, high fecundity, and short generation time. The fly genome contains about 14,000 genes and many are well-conserved in mammals (Bier, 2005). Furthermore, it would avoid the ethical controversy if using fruit flies as human disease model. In addition, fly as a fast-track model could be used for screening novel compounds from the large chemical libraies, which will shorten the time from experimental setup to clinical use.

Despite the conservation of important basic cell processes in flies and mammals, there are still some differences between flies and mammals. Flies have some limitations in the study of intestinal inflammatory disease. First of all, due to differences in physiology and development, it is difficult to directly apply the results of *Drosophila* intestinal microbiota to mammals. Second, there are the extensive anatomical differences in gut between flies and mammals. Flies lack specific vertebrate cell types such as goblet cells, tuft cells, paneth cells, and M cells. Third, immune systems are different between mammalian and fly. *Drosophila* does not have the acquired immune system found in mammals, and solely depends on general mechanisms of innate immunity for its immune defenses (Lemaitre and Hoffmann, 2007). However, flies have high conserved features for innate immunity with mammals, such as immune cascades, signal transduction pathways, and transcriptional regulators (Arch et al., 2022).

In general, flies offer value as parallel alternatives to mammal models in use for screening drugs that treat IBD. Its potential as intestinal inflammatory disease research model is important for discovering mechanisms of intestinal disease and potential therapeutics.
